# The Improvement of Hyperglycemia after RYGB Surgery in Diabetic Rats Is Related to Elevated Hypothalamus GLP-1 Receptor Expression

**DOI:** 10.1155/2016/5308347

**Published:** 2016-08-25

**Authors:** Jazyra Zynat, Yuyu Guo, Yingli Lu, Dongping Lin

**Affiliations:** Department of Endocrinology and Metabolism, Shanghai Ninth People's Hospital, Shanghai Jiaotong University School of Medicine, Shanghai, China

## Abstract

*Objectives*. This study aimed to explore the expression of GLP-1 receptor in hypothalamus and gastrointestinal tissues after Roux-en-Y gastric bypass (RYGB) surgery in diabetic rats.* Methods*. Male 12-week-old Wistar rats (control) and Goto-Kakizaki rats (diabetic) were randomly divided into two groups, respectively: control sham surgery group (C), control RYGB group (C + R), diabetic sham surgery group (D), and diabetic RYGB group (D + R). Body weight and blood glucose were monitored before and after surgery every week. Eight weeks after surgery, all rats were sacrificed and the serum fasting GLP-1 concentrations were measured by ELISA. GLP-1R and DPP-4 expression in hypothalamus and ileum were measured by RT-PCR.* Results*. The body weight and fasting/random blood glucose in the D + R group decreased significantly compared with the D group (*P* < 0.05). Serum GLP-1 levels in diabetic rats treated with RYGB were higher than the corresponding sham surgery rats. The expression of GLP-1R of hypothalamus in RYGB-treated diabetic rats was significantly higher than those of the sham surgery diabetic rats and both control group rats (*P* < 0.05). We found a negative correlation between hypothalamus GLP-1R mRNA and blood glucose level. No significant difference was seen in ileum GLP-1R and DPP-4 expression among all groups.* Conclusions*. RYGB efficiently promoted serum GLP-1 levels and the expression of GLP-1 receptor in the hypothalamus in diabetic rats. These data suggest that the hypothalamus GLP-1R may play an important role in the GLP-1 system for improving glucose homeostasis after reconstruction of the gastrointestinal tract.

## 1. Introduction

Roux-en-Y gastric bypass (RYGB) has a rapid and drastic effect in type 2 diabetic patients, many of whom show complete remission within days of the procedure [1, 2]. A number of studies have demonstrated that gut hormones, especially GLP-1, may play a pivotal role in the regulation of glucose metabolism in response to this surgery [3, 4]. GLP-1 receptor was first found in pancreatic beta cells [5]. Activated GLP-1R stimulates the adenylyl cyclase pathway which results in increased insulin synthesis and release of insulin. Subsequently, GLP-1 receptors are known to be expressed in a wide range of tissues, including lung, heart, kidney, stomach, intestine, pituitary, skin, and nodose ganglion neurons of the vagus nerve, as well as the hypothalamus, nucleus of the tractus solitaries, and reticular nucleus in the central nervous system (CNS) [6]. However, the gene expression and functionality of CNS GLP-1 receptor (GLP-1R) remain largely unknown in diabetes after RYGB surgery.

In our study, we explored the impact of RYGB on GLP-1R expression in hypothalamus and ileum tissues in both diabetic and nondiabetic rats, aimed to identify the effect of GLP-1 receptor on glucose metabolism, and uncover the mechanisms behind the RYGB surgery after RYGB.

## 2. Materials and Methods

### 2.1. Experimental Animals

Male 12-week-old Wistar and Goto-Kakizaki (GK) rats were purchased from SLACK Laboratories, SIBS, Shanghai, China. Animals were housed at an ambient temperature of 22 ± 2°C and maintained under a normal 12 hr light/dark cycle with free access to food and water. Rats were allowed 2 weeks for acclimation before the start of experiments. Wistar rats (*n* = 12) were randomly assigned to a RYGB surgery of control rats (C + R, *n* = 6) and sham surgery of control rats (C, *n* = 6). Goto-Kakizaki rats (*n* = 20) were also randomly assigned to RYGB surgery of diabetic rats (D + R, *n* = 14) and sham surgery of diabetic rats (D, *n* = 6). Six rats died within three days after RYGB surgery. All the animal procedures were performed in accordance with the ethical principles in animal research adopted by the Department of Laboratory Animal Science, Jiaotong University School of Medicine, Shanghai, China.

### 2.2. Surgery Procedure

After overnight fasting, rats were anesthetized with chloral hydrate (3.5 mL/kg). A dose of 200 mg/kg penicillin sodium (Shanghai Xinya Pharmaceutical Co. Ltd.) was given intravenously as prophylactic antibiotic. Midline laparotomy was performed under sterile conditions. Gastric bypass surgery was performed as previously described [7] and in the sham surgery group there was only an anterior abdominal wall incision which was immediately sutured without disturbing the internal organs. After surgery, animals were housed individually, and body weight and blood glucose were monitored daily. To allow the surgical anastomosis to heal, animals were fasting and only fed with water for the three days after surgery (days 1, 2, and 3).

### 2.3. Measurement of Blood Glucose, Body Weight, and Serum GLP-1 Levels

Blood glucose was detected weekly by the electronic glucometer (Siemens Healthcare Diagnostics Inc.). At 2, 5, and 8 weeks after surgery, blood glucose was detected at the overnight fasting condition. One week before surgery and at 3, 4, 6, and 7 weeks, random blood glucose was detected. Body weight was measured using an electronic balance weekly.

Eight weeks after surgery, tail blood was obtained to assess the total GLP-1 levels; the tubes for blood samples of GLP-1 contained 20 *μ*L dipeptidyl dipeptidase IV inhibitor, measured at room temperature by using ELISA kits (Shanghai Westang).

### 2.4. Determination of GLP-1R and DPP-4 mRNA in Tissues with Real-Time PCR

Hypothalamus and ileum samples were separated after rats were sacrificed. Hypothalamus was separated according to previous study [8]; perpendicular to the midline suture, a cut was made at the septopalliomesencephalic tract and at the third cranial nerves. At 2.0 mm parallel to the midline, two cuts were made and finally a cut from the anterior commissure to 1.0 mm ventral to the posterior commissure was made. Ileum tissue was separated at the site about 10 cm away from the abovementioned proximal-distal jejunum end by side anastomosis. Total RNA was prepared from tissue mentioned above using a TRIzol reagent (TIANGEN) according to the manufacturer's instructions, after which the RNA quantity and purity were evaluated by a model ND-2000 apparatus (Thermo Scientific NanoDrop 2000, USA). The integrity of the RNA was confirmed by agarose-formaldehyde gel electrophoresis. Using cDNA Reverse Transcription Kit (Promega Corporation, USA), first-strand cDNA was synthesized from individual samples in 20 *μ*L reactions from 200 ng of total RNA following the manufacturer's instructions. The integrity of the cDNA was confirmed by amplifying *β*-actin. The real-time PCR was conducted by a LightCycler 96 (Roche Applied Science, Switzerland) employing SYBR Green I as the dsDNA specific binding dye for continuous fluorescence monitoring. The PCR protocol comprised 5 min at 95°C and 45 cycles of 15 s at 95°C, 15 s at 58°C, and 15 s at 72°C. The sequences of the GLP-1R primers are as follows: forward 5′-AGT AGT GTG CTC CAA GGG CAT-3′, reverse 5′-AAG AAA GTG CGT ACC CCA CCG-3′; and *β*-actin forward 5′-GCC CCT CTG AAC CCT AAG-3′, reverse 5′-CAT CAC AAT GCC AGT GGT A-3′; DPP-4 forward 5′-CAA ATC ACC GCT CCT GCA T-3′, reverse 5′-TCA TAG TCG CAG ATC GCC A-3′. The mRNA levels of GLP-1R were compared by calculating the crossing point (Cp) value and normalized by the reference genes (*β*-actin).

### 2.5. Statistical Analysis

Measurement data were expressed as the mean ± SD. The data were analyzed with a repeated-measures one-way ANOVA. *P* < 0.05 was considered statistically significant. All statistical analyses were performed using SPSS 19.0 statistical software.

## 3. Results

### 3.1. Effects of RYGB on Body Weight and Blood Glucose

**Figure 1:**
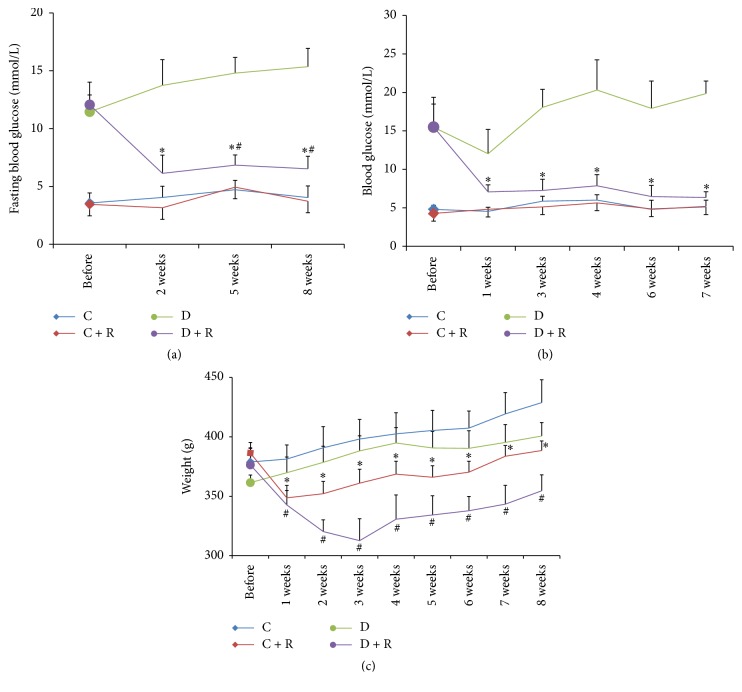
(a) Fasting blood glucose of four groups. ^*∗*^ *P* < 0.01 compared with D group; ^#^ *P* < 0.01 compared with C group. (b) Random blood glucose of four groups. ^*∗*^ *P* < 0.01 compared with D group. (c) The change of weight in each study group was assessed before and every week after surgery. ^*∗*^ *P* < 0.01 compared with C group; ^#^ *P* < 0.01 compared with D group.

Prior to the surgery, there were no significant differences in body weight and random/fasting blood glucose levels between the C and C + R groups or the D and D + R groups of rats. The two RYGB-treated groups exhibited a significant reduction in the mean body weight for the first three weeks after surgery (*P* < 0.01) and then increased gradually. The fasting blood glucose and random blood glucose in the D + R group decreased significantly compared with the D group after surgery (*P* < 0.05). However, no significant difference in fasting/random blood glucose was seen between C and C + R groups (Figure 1).

### 3.2. Serum GLP-1 Levels

**Figure 2:**
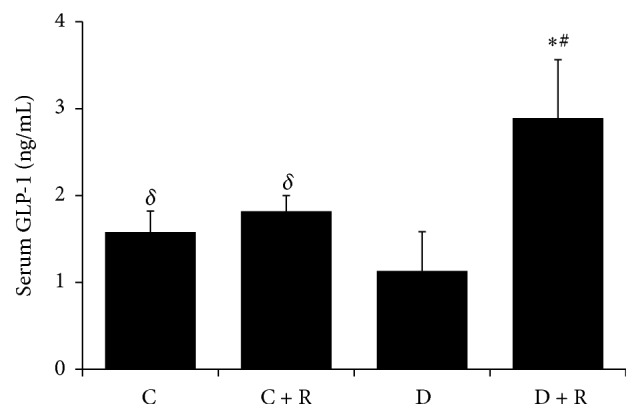
Serum GLP-1 level in four groups. ^*∗*^ *P* < 0.01 compared with D group; ^#^ *P* < 0.01 compared with C group; ^*δ*^ *P* < 0.05 compared with D group.

Eight weeks after surgery, serum GLP-1 concentrations were less in diabetic rats (*P* < 0.05) than control rats, whereas GLP-1 concentrations were greater in RYGB-treated diabetic rats (*P* < 0.01) than group D rats. The GLP-1 concentrations were not significantly different between the C + R and C group (Figure 2).

### 3.3. GLP-1R and DPP-4 mRNA Expression in Tissues

**Figure 3:**
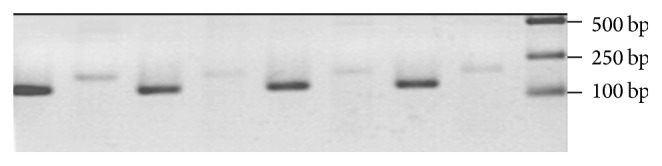
The expression of GLP-1R mRNA in the hypothalamus in four groups. From right to left: marker, C GLP-1R, C *β*-actin, C + R GLP-1R, C + R *β*-actin, D GLP-1R, D *β*-actin, D + R GLP-1R, and D + R *β*-actin. The expression of GLP-1 receptor of hypothalamus in RYGB-treated GK rats was significantly higher than all other groups.

**Figure 4:**
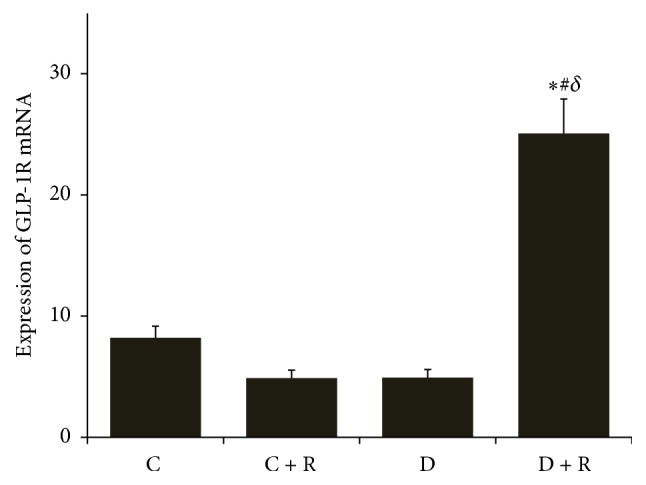
The crossing point (Cp) value of GLP-1R mRNA in the hypothalamus by RT-PCR in four groups. ^*∗*^ *P* < 0.01 compared with D group; ^#^ *P* < 0.05 compared with C group; ^*δ*^ *P* < 0.01 compared with C + R group.

The expression of GLP-1R mRNA in hypothalamus of D + R group was increased significantly compared with all other study groups of rats (*P* < 0.05). We found a negative correlation between hypothalamus GLP-1R mRNA and blood glucose level. There was no statistical difference in GLP-1R mRNA between D group, C group, or C + R groups (*P* > 0.05) (Figures 3 and 4). The expressions of GLP-1R and DPP-4 mRNA in ileum were not significantly different between groups.

## 4. Discussion

Accumulating evidence has shown that Roux-en-Y gastric bypass (RYGB) surgery is one of the most effective therapies to achieve sustained improvement in T2DM patients [3, 9, 10]. One of the most important mechanisms of the antidiabetic effect was regarded as RYGB surgery changes in gastrointestinal hormone secretions. RYGB promotes the glucoincretin hormone GLP-1 secretion, which, in turn, stimulates insulin secretion, gastric restriction, and malabsorption [11], enhances delivery of nutrients to the ileum [12], and increases energy expenditure and/or other metabolic adaptations [13]. In this study, we observed the expected improvement of blood glucose and increased GLP-1 in Roux-en-Y gastric bypass treated diabetic rats. The main finding of our study is that the hypothalamus GLP-1R expressions in RYGB-treated diabetic rats were significantly higher than those of the sham surgery diabetic rats. We also found a negative correlation between hypothalamus GLP-1R expression and blood glucose level. Taken together, these data suggest that the hypothalamus GLP-1R may play an important role in the GLP-1 system for improving glucose homeostasis after reconstruction of the gastrointestinal tract.

GLP-1R is widely expressed in various tissues. Given the fact that the plasma half-life of the intact GLP-1 peptide is short in vivo [14], the GLP-1R agonist, exendin-4, is considered to be a more potent stimulator of insulin secretion [15]. A recent study showed reduced GLP-1R expression in diabetic rats and downregulation of GLP-1R by high glucose in RIN-m5F cells [16], implying that decreased GLP-1 receptor level may inhibit the bioactivity of GLP-1 and thus contribute to the progression of T2DM. In the present study, the increased blood GLP-1 concentration and upregulated GLP-1 receptor expression were observed in rat hypothalamus after RYGB surgery (*P* < 0.05, compared to that of the control group rats). These findings demonstrated that RYGB surgery could increase the levels of GLP-1 and GLP-1R in the diabetic rats, implying that RYGB may improve glucose homeostasis by upregulating GLP-1/GLP-1R expressions. We also found a negative correlation between hypothalamus GLP-1R expression and blood glucose level. This is an interesting finding, which, for the first time, showed evidence that hypothalamus GLP-1R was involved in the glucose regulation after RYGB, directly or indirectly. These results demonstrated the potential role of GLP-1/GLP-1R in high glucose environment.

However, no significant difference in ileum expression of GLP-1R was seen after RYGB from real-time PCR analysis. The reason that ileum GLP-1R did not change is unknown yet. One possibility is that the GLP-1R protein level, which was not measured in this study, may have changed after RYGB. Another possibility is that the expression of GLP-1R in other tissues may play a role in glucose regulation. A recent research found that GLP-1R expression is reduced in gastric glands from T2DM patients [17]. The expression of GLP-1R in other tissues and its role in glucose regulation after RYGB need more exploration in the future.

Dipeptidyl peptidase-4 (DPP-4), which has been proposed as a target for pharmacological intervention in patients with type 2 diabetes [18], was primarily found not only in the brush-border membranes of the kidney and the small intestine but also in other tissues (hepatocytes around bile canaliculi, epithelial cells of the pancreas, and in capillary endothelium) and in a soluble form in plasma [19]. Previous studies have shown that the fasting DPP-4 activity decreases in parallel with the rise in incretin levels [20]. The decrease in DPP-4 activity was rather modest compared to the robust increase in incretin levels [21]. Circulating DPP-4 activity is significantly elevated in type 1 and type 2 diabetics, which could contribute to the reduction in circulating active GLP-1 and to the consequent hyperglycemia [22]. DPP-4 is mainly a tissue enzyme, and the relationship between tissue and circulating DPP-4 activity is unknown. We hypothesized that GLP-1 levels elevation observed after RYGB surgery would be accounted for, in part, by decreased intestinal DPP-4 expression. The present study showed that no significant difference in ileum DPP-4 expression was detected between diabetic rats and matched healthy control rats. However, We cannot exclude the role of DPP-4 in the glucose regulation after RYGB yet. One reason is that we did not measure the tissue or circulating DPP-4 enzyme activity. Another reason is that the DPP-4 mRNA expression and enzyme activity in other tissues, just like GLP-1R we mentioned above, are still unknown. The DPP-4 mRNA expression and enzyme activity in other tissues and its role in glucose regulation need future investigation.

In conclusion, our data illustrated a negative correlation between hypothalamus GLP-1R mRNA and blood glucose level after RYGB surgery. The upregulated GLP-1R expression in hypothalamus may contribute to the regulation of glucose homeostasis. This research can provide ideas and information for research to further study the GLP-1 receptor-associated signaling pathways regulation and GLP-1 receptor expression in CNS. Further research is needed to evaluate the potential beneficial effects of RYGB surgery, the mechanism behind the tissue receptor regulation, and further role in the therapeutic range.

## Abbreviation

RYGB: Roux-en-Y gastric bypass
GK: Goto-Kakizaki
T2DM: Type 2 diabetes mellitus
GLP-1: Glucagon Like Peptide-1
GLP-1R: Glucagon Like Peptide-1 Receptor
DPP-4: Dipeptidyl peptidase-4
ELISA: Enzyme linked immunosorbent assay.
